# PD-1 inhibitor-induced rheumatic, endocrine, and sarcoidosis-like immune-related adverse events in metastatic melanoma are associated with improved survival and lower corticosteroid exposure

**DOI:** 10.1093/immadv/ltag004

**Published:** 2026-02-21

**Authors:** Maria Lindén, Hifaa Al Remawi, Anna Fager, Levent M Akyürek, Anna Rudin, Lars Ny, Sara Bjursten, Ankur Pandita, Max Levin

**Affiliations:** Department of Oncology, Institute of Clinical Sciences, Sahlgrenska Academy, University of Gothenburg, Gothenburg, Sweden; Department of Oncology, Sahlgrenska University Hospital, Gothenburg, Sweden; Department of Molecular and Clinical Medicine/Wallenberg Laboratory, Institute of Medicine, Sahlgrenska Academy, University of Gothenburg, Gothenburg, Sweden; Department of Oncology, Institute of Clinical Sciences, Sahlgrenska Academy, University of Gothenburg, Gothenburg, Sweden; Department of Oncology, Sahlgrenska University Hospital, Gothenburg, Sweden; Department of Oncology, Institute of Clinical Sciences, Sahlgrenska Academy, University of Gothenburg, Gothenburg, Sweden; Department of Oncology, Sahlgrenska University Hospital, Gothenburg, Sweden; Department of Clinical Pathology, Sahlgrenska University Hospital, Gothenburg, Sweden; Department of Laboratory Medicine, Institute of Biomedicine, Sahlgrenska Academy, University of Gothenburg, Gothenburg, Sweden; Department of Rheumatology and Inflammation Research, Institute of Medicine, Sahlgrenska Academy, University of Gothenburg, Gothenburg, Sweden; Department of Oncology, Institute of Clinical Sciences, Sahlgrenska Academy, University of Gothenburg, Gothenburg, Sweden; Department of Oncology, Sahlgrenska University Hospital, Gothenburg, Sweden; Department of Oncology, Institute of Clinical Sciences, Sahlgrenska Academy, University of Gothenburg, Gothenburg, Sweden; Department of Oncology, Sahlgrenska University Hospital, Gothenburg, Sweden; Department of Oncology, Institute of Clinical Sciences, Sahlgrenska Academy, University of Gothenburg, Gothenburg, Sweden; Department of Oncology, Sahlgrenska University Hospital, Gothenburg, Sweden; Department of Molecular and Clinical Medicine/Wallenberg Laboratory, Institute of Medicine, Sahlgrenska Academy, University of Gothenburg, Gothenburg, Sweden; Department of Oncology, Institute of Clinical Sciences, Sahlgrenska Academy, University of Gothenburg, Gothenburg, Sweden; Department of Oncology, Sahlgrenska University Hospital, Gothenburg, Sweden; Department of Molecular and Clinical Medicine/Wallenberg Laboratory, Institute of Medicine, Sahlgrenska Academy, University of Gothenburg, Gothenburg, Sweden

**Keywords:** PD-1 inhibitor, melanoma, immune-related adverse events, survival

## Abstract

**Introduction:**

Programmed cell death protein 1 (PD-1) inhibitors improve survival in advanced melanoma but can induce immune-related adverse events (irAEs). IrAEs have been linked to better outcomes. However, it remains unclear whether specific irAE types drive this effect and how corticosteroid treatment of irAEs influences survival.

**Materials and methods:**

A seven-year retrospective cohort study of 301 patients with advanced cutaneous melanoma treated with single-agent PD-1 inhibition at Sahlgrenska University Hospital. irAEs were identified using CTCAE v4.0/v5.0, and irAEs requiring systemic corticosteroids or endocrine replacement therapy were included. Corticosteroid therapy was categorized as low dose (≤0.5 mg/kg prednisolone equivalent) or high dose (>0.5 mg/kg). Overall survival (OS) was assessed using Kaplan–Meier and Cox models, including time-dependent analyses to address immortal time bias.

**Results:**

Patients with irAE (109 of 301 patients) had longer OS than those without irAEs. Of the eight most common irAEs, four were associated with superior survival, one was borderline significant, and three were non-significant. Rheumatic irAEs and late-onset thyroid irAEs remained associated with improved OS after adjustment for negative prognostic factors and immoral time bias. Colitis irAE were borderline significant in univariate analysis. Sarcoidosis-like and hypophysitis irAEs were rare but conferred excellent outcomes. Hepatitis, nephritis, and pneumonitis were not associated with better survival. Most survival-associated irAEs were treated with a lower start dose of corticosteroids but duration and time to onset were similar to non–survival-associated irAEs.

**Conclusion:**

Rheumatic, endocrine, and sarcoidosis-like irAEs are markers of superior survival and suggest that lower initial corticosteroid doses may preserve PD-1 inhibitor efficacy.

## Graphical Abstract

**Graphical Abstract:**
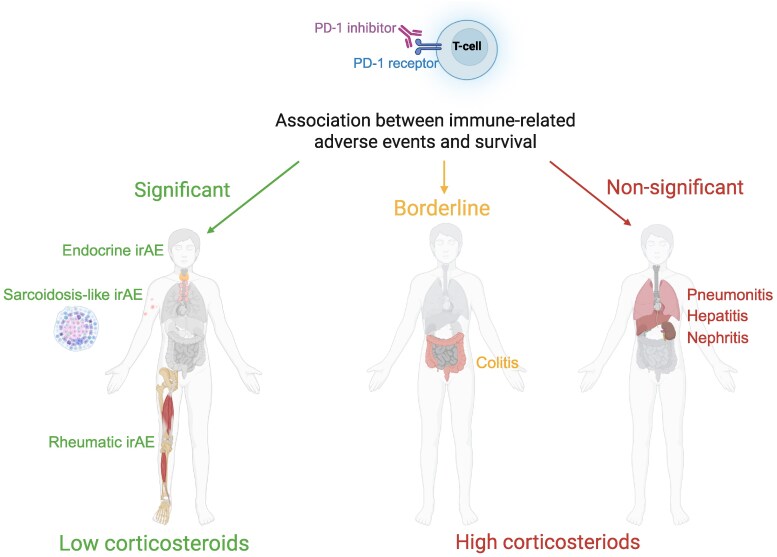

## Introduction

Immune checkpoint inhibitors (ICI) have improved survival outcomes for many types of cancer, including metastatic melanoma [1–4]. The most clinically important immune checkpoint is programmed cell death protein 1 (PD-1). PD-1 is an inhibitory receptor on T cells and binding of its ligands, PD-L1 or PD-L2, causes T-cell inactivation. Blocking PD-1/PD-L1 binding with inhibitory antibodies, such as nivolumab or pembrolizumab, activates T cells and promotes an antitumor response in some patients. Although immunotherapy with PD-1 inhibitors has improved survival rates for patients with melanoma, many do not respond to treatment. Therefore, it is important to investigate why some patients display better responses, in the hopes of converting non-responders into responders in the future.

ICI-induced activation of T cells may also damage healthy tissue, causing a set of autoimmune adverse events known as immune-related adverse events (irAE) [5]. These are a diverse group of immunological reactions that can affect any organ, ranging from mild rashes to potentially fatal toxicities such as myocarditis [6]. Interestingly, several studies have found that patients with irAEs have a better prognosis than those without [7]. However, the association between specific types of irAEs and survival remains understudied. In this study, we examined the relationship between the eight most prevalent PD-1 inhibitor-induced irAEs and survival outcomes in patients with advanced cutaneous melanoma. In addition, we compared the start dose, time to onset, and duration of corticosteroid treatment between survival-associated and non-survival-associated irAEs.

## Materials and methods

### Study design

This is a seven-year retrospective analysis of 301 patients with unresectable stage III (M0) or stage IV (M1a-c) cutaneous melanoma who were treated with single PD-1 inhibitor agents at the Department of Oncology at Sahlgrenska University Hospital in Gothenburg, Sweden between 1 September 2015, and 7 July 2022. The department treats patients from western Sweden, which has a total population of 1.8 million. Patient data were retrieved from electronic medical records. All patients over 18 years of age who received at least one dose of PD-1 inhibitor treatment during the study period were included, while those who received treatment with CTLA-4 inhibitors at any point during the time frame were excluded. Patients with brain metastases (M1d) were also excluded since this group of patients only occasionally receive PD-1 inhibitor therapy. PD-1 inhibitor therapy consisted of the intravenous administration of nivolumab (3 mg/kg or 240 mg every two weeks, or 6 mg/kg or 480 mg every four weeks) or pembrolizumab (2 mg/kg or 200 mg every three weeks, or 4 mg/kg or 400 mg every six weeks). Patients received treatment until they experienced clinical or radiological progression or developed serious adverse events, or for a maximum of two years.

### Ethical considerations

All research was performed in accordance with the Declaration of Helsinki of the World Medical Association. The research protocol was approved by the Swedish Ethical Review Authority (approval number 477-18). The ethics committee waived the requirement for informed consent because the data are presented at a group level.

### Baseline characteristics

The following baseline characteristics were collected for each patient: age; sex; body mass index (BMI); Eastern Cooperative Oncology Group (ECOG) performance status; TNM stage at diagnosis (according to the eighth edition of the American Joint Committee on Cancer (AJCC) staging manual); BRAF mutation status; and blood levels of lactate dehydrogenase (LDH) and C-reactive protein (CRP), as well as whether or not patients had received any prior systemic cancer therapies. BRAF mutations were BRAF_V600E, BRAF_V600K, and BRAF_V600R; other mutations were defined as wild type (WT). The upper limit of normal (ULN) for LDH was defined as 3.4 µkat/L. Data were collected on the start date of treatment, date of death, time on treatment, and reason for treatment discontinuation.

### Immune-related adverse events

The occurrence and severity of irAE were diagnosed by the treating physician or nurse according to the Common Terminology Criteria for Adverse Events (CTCAE) v. 4.0 (before 27 November 2017) and v5.0. Sarcoidosis-like irAE were diagnosed based on inflammatory fluorodeoxyglucose (FDG)-uptake on positron emission tomography (PET) scans in combination with sarcoidosis-like non-caseating granulomas in biopsies from affected tissue as determined by an experienced pathologist [8, 9]. Medical treatment with corticosteroids (and other immunosuppressive drugs) or endocrine replacement therapy was initiated according to European Society for Medical Oncology (ESMO) guidelines [5, 10]. The survival analysis included irAEs that required immune-modulating treatment or endocrine hormone replacement therapy. Depending on the irAE type, this corresponds to irAE grades 2–4 according to the CTCAE.

In addition to irAE type and severity, corticosteroid exposure was systematically recorded. For each irAE requiring systemic corticosteroids, the start date, initial corticosteroid dose (expressed as mg/kg of prednisolone equivalent), duration of treatment, and need for second-line immunosuppressive therapy were documented. Corticosteroid exposure was stratified into low-dose (≤0.5 mg/kg) and high-dose (>0.5 mg/kg) categories based on the starting dose. For endocrine irAEs, corticosteroid replacement (e.g. hydrocortisone for hypophysitis) was not classified as immunosuppression.

### Statistical analyses

The primary endpoint was overall survival (OS) in relation to baseline characteristics and to presence or absence of irAE, defined as the number of days from treatment start to either death or censoring date. The secondary endpoint was to investigate the impact of immune-modulating treatment on survival outcomes. A separate exploratory analysis examined the relationship between corticosteroid exposure and overall survival. Survival outcomes were compared between patients treated with low- and high-dose corticosteroids and those who did not receive corticosteroids (no irAE). The impact of corticosteroid dose, time to start, and duration of corticosteroids was evaluated using Kaplan–Meier and Cox proportional hazards models.

Baseline characteristics were categorized to conduct the analysis. For baseline analysis, we included: sex (male vs. female), age (< 65 vs. ≥ 65 years), BMI (< 25 vs. ≥ 25), ECOG performance status (0-1 vs. 2–4), stage (M0-M1b vs. M1c), baseline LDH (< ULN vs. ≥ ULN), baseline CRP (< 5 vs. ≥ 5 mg/L), previous treatment (yes or no), and activating BRAF mutation (yes or no). The cutoff points of these variables were chosen based on previous studies reporting baseline characteristics [2, 11] except for CRP, where the lab reference interval was used to determine the cutoff point. Patients with missing data for specific baseline characteristics, e.g. unknown baseline CRP, were excluded from the statistical analysis considering that specific variable.

Differences in median OS were visualized using Kaplan–Meier curves and assessed through hazard ratios (HR) of death, calculated using the Cox proportional hazard model. The log-rank (Mantel–Cox) test was assessed to determine the statistical significance of survival differences between groups.

The patient cohort was divided into two groups: those with irAE and those without. Patients with irAE was further divided into each specific irEA and compared to the ‘no irAE’ group. For the initial univariate analysis between the two groups (irAE vs ‘no irAE’), and between each specific irAE vs ‘no irAE’, Kaplan–Meier curves were used to visualize differences in median OS and assessed through HR, calculated using Cox regression, and the log-rank test to determine the statistical significance of survival difference. Multivariate Cox regressions were assessed to adjust the HR for negative prognostic baseline factors, ensuring 10 events per variable, and adjusted *P*-values were calculated using Walds-test. Lastly, time-dependent Cox regression was applied to account for immortal time bias. In this analysis, irAE status was treated as a time-dependent variable, which allowed patients to transition from the ‘no irAE’ group to the ‘irAE’ group when they developed an irAE. HR was calculated using the Cox proportional hazards model, and the Schoenfeld residual method was used to assess the validity of the Cox proportional hazards assumption. When the proportional hazards assumption was violated, as indicated by a significant test result (*P* < 0.05), the population was split into two groups to observe how the HR changes over time. Baseline HRs were reported along with a description of how HR changed over time. Adjusted *P*-values were calculated using Walds-test.

Statistically significant results were defined as 95% confidence intervals (CIs) for OS and HR, and *P*-values of 0.05. R version 4.4.2 and GraphPad Prism 10.4.0 were used for statistical analysis, and professional biostatisticians assisted with all analyses.

## Results

### Survival outcomes in patients with different baseline characteristics

**Figure 1:**
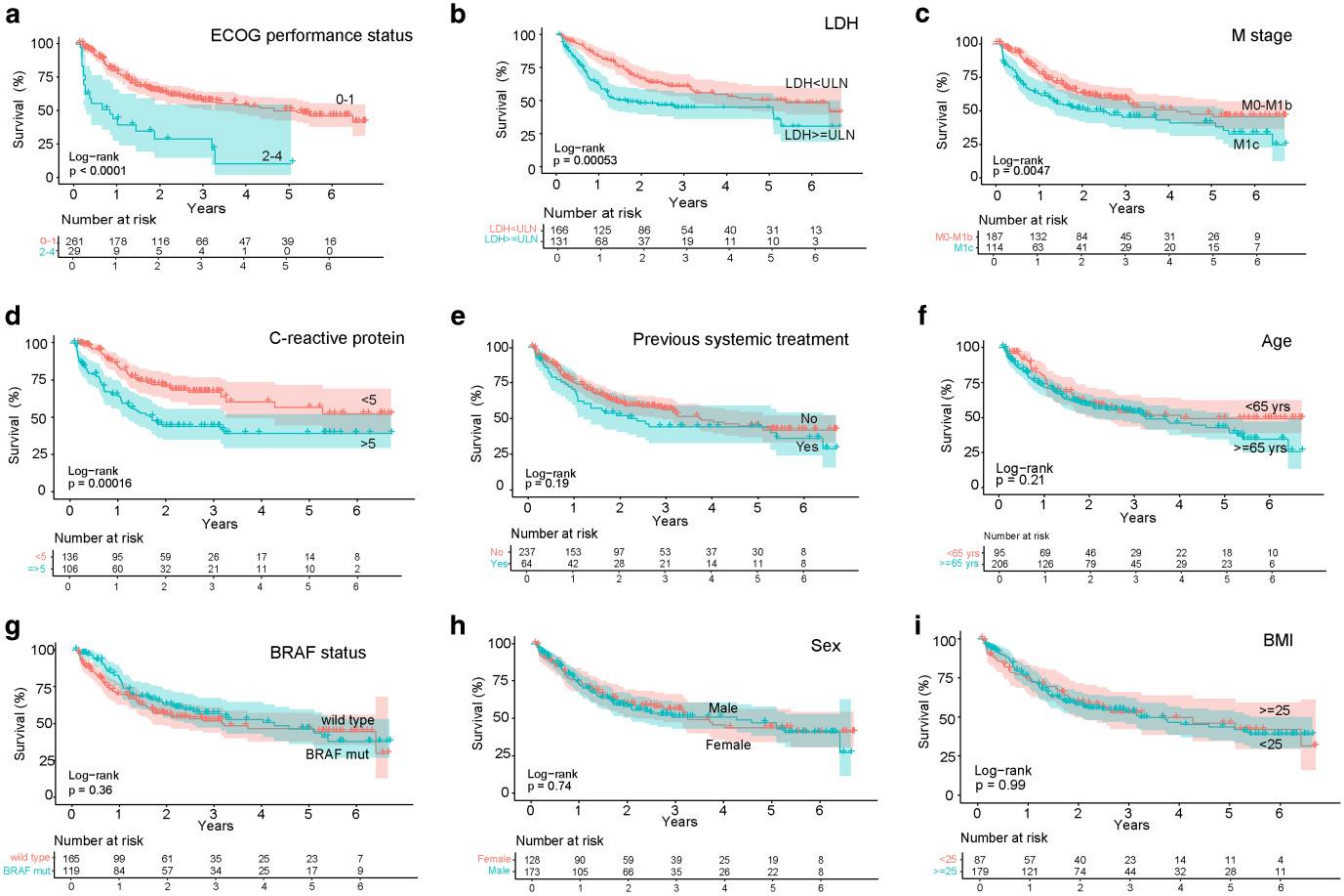
Association of baseline characteristics with survival. ECOG performance status ≥2 (a), baseline LDH ≥ ULN (b), advanced clinical stage (M1c vs M1a-b) (c), and baseline CRP ≥ 5 mg/ml (d) were associated with significantly impaired survival. Previous systemic treatment (yes vs no) (e), age (<65 vs ≥65 years) (f), BRAF-status (mutant vs wild type) (g), sex (male vs female) (h), and BMI (<25 vs ≥25) (i) were not associated with survival outcomes. The adjusted *P*-value was calculated using the Wald test. ECOG: Eastern Cooperative Oncology Group, LDH: lactate dehydrogenase, ULN: upper limit of normal (3.4 µkat/L), CRP: C-reactive protein.

A total of 301 patients were included in the analysis, and their baseline characteristics are presented in Supplementary Table S1. The median follow-up period (i.e. time from treatment start to death or censoring) was 20 months. Three- and five-year survival rates for all patients were 53% and 45%, respectively (Supplementary Figure S1). The following baseline characteristics were significantly associated with decreased OS (Fig. 1a–d): ECOG performance status ≥2 (hazard ratio [HR]: 3.35, 95% confidence interval [CI]: 2.09–5.38, *P* < 0.0001), baseline LDH ≥ ULN (HR: 1.83, 95% CI: 1.29–2.58, *P* = 0.00053), stage M1c (HR: 1.62, 95% CI: 1.16–2.28, *P* = 0.0047), and increased baseline CRP (≥5 mg/L) (HR: 2.15, 95% CI: 1.43–3.24, *P* = 0.00016). Previous systemic antitumor treatment (*P* = 0.19), age (*P* = 0.21), BRAF status (*P* = 0.36), sex (*P* = 0.74), and BMI (*P* = 0.99) were not associated with survival outcomes (Fig. 1e–i).

Patients with missing baseline data on ECOG (*n* = 11), LDH (*n* = 4), CRP (*n* = 59), BMI (*n* = 35), or BRAF status (*n* = 17) were excluded from analyses regarding those specific variables.

### Immune-related adverse events

**Figure 2:**
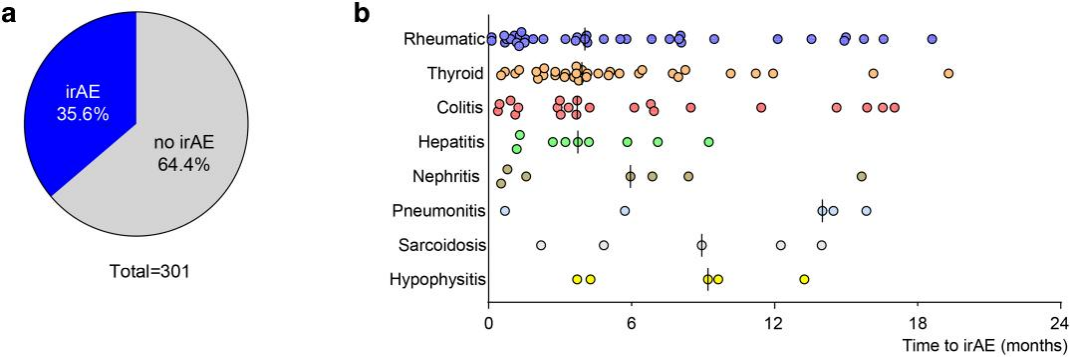
Frequency and time to onset for the eight most common irAEs. (a). Frequency of irAE. (b). Time to irAE onset for the eight most common irAEs. The type of irAE is indicated at the y axis and each dot represents one patient. The black lines represent median time to debut. irAE: immune-related adverse events.

A total of 109 out of 301 patients (35.6%; Fig. 2a) developed at least one irAE that required immune-modulating treatment or endocrine hormone replacement therapy during the study period. The median time to irAE onset was 125 days (interquartile range [IQR] 61–253 days). The time to onset and frequency of the eight most common irAEs are shown in Fig. 2b. The four most common irAE were rheumatic (12.3%, 37/301), thyroid (11.0%, 33/301), colitis (7.0%, 21/301), and hepatitis (3.0%, 9/301), followed by nephritis (2.3%, 7/301), pneumonitis (1.7%, 5/301), sarcoidosis-like irAE (1.7%, 5/301), and hypophysitis (1.7%, 5/301).

### Survival outcomes in patients experiencing common immune-related adverse events

**Figure 3:**
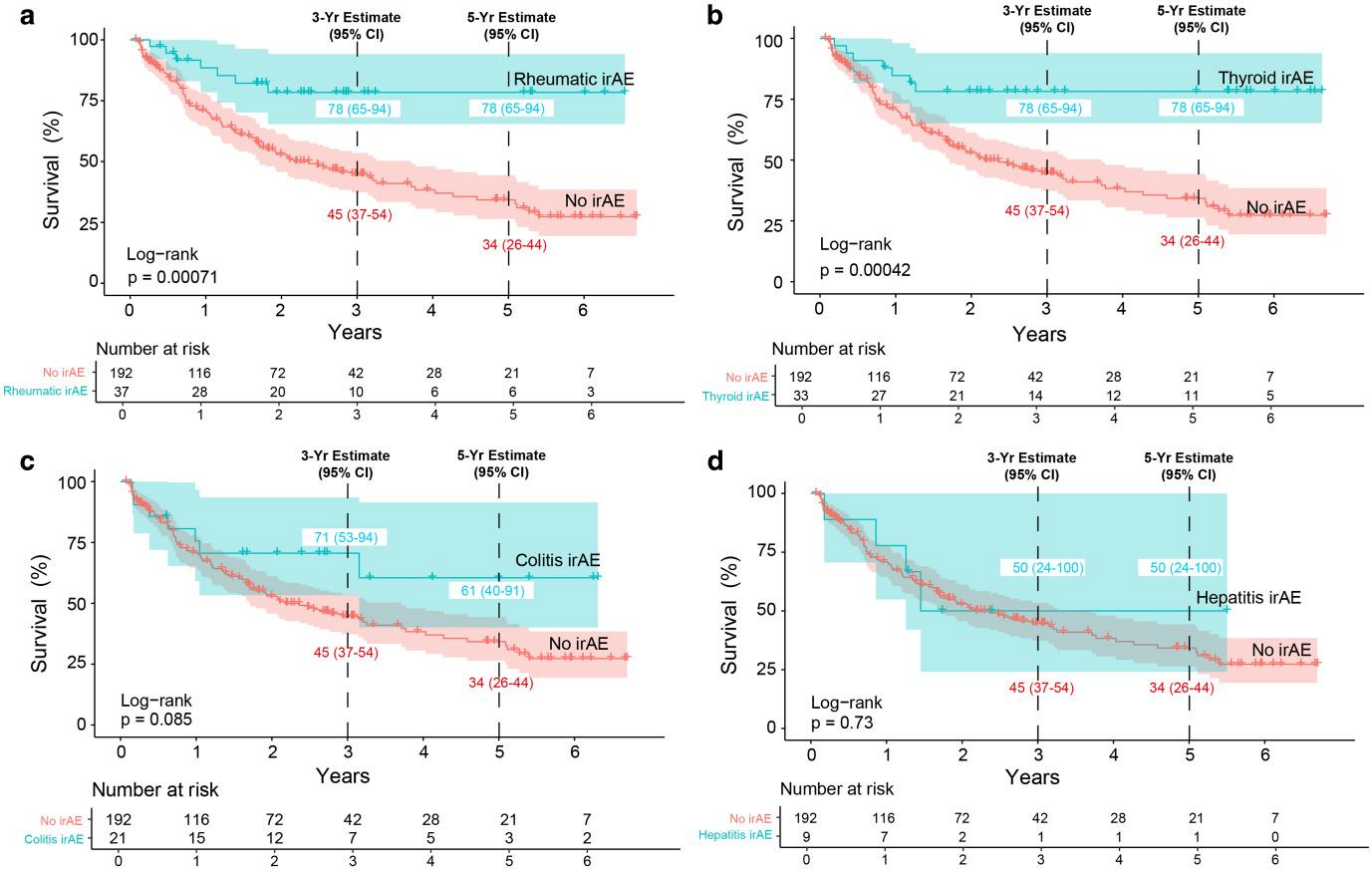
Survival-associations in common irAEs. Patients with rheumatic irAE (a) and thyroid irAE (b) had superior survival compared to patients with no irAE whereas patients with colitis irAE (c) and hepatitis irAE (d) did not. *P*-values were calculated with the log-rank test, where *P* < 0.05 was considered statistically significant. Confidence intervals (CI) for median survival were set to 95%. 3- and 5- year-survival with confidence intervals are indicated in the graphs. irAE: immune-related adverse events.

Patients with any irAE had a superior median OS and a lower risk of death than patients without irAE (HR: 0.49, 95% CI: 0.33–0.72, *P* = 0.00024). Separate analysis of individual irAE showed that patients with rheumatic irAE (HR: 0.29, 95% CI: 0.13–0.62, *P* = 0.00071, Fig. 3a) and thyroid irAE (HR: 0.27, 95% CI: 0.13–0.59, *P* = 0.00042, Fig. 3b) had a statistically significant longer OS and patients with colitis had borderline significant longer OS (HR: 0.52, 95% CI: 0.24–1.11, *P* = 0.085, Fig. 3c). Hepatitis irAE was not associated with superior survival (HR: 0.84, 95% CI: 0.31–2.27, *P* = 0.73, Fig. 3d).

**Figure 4:**
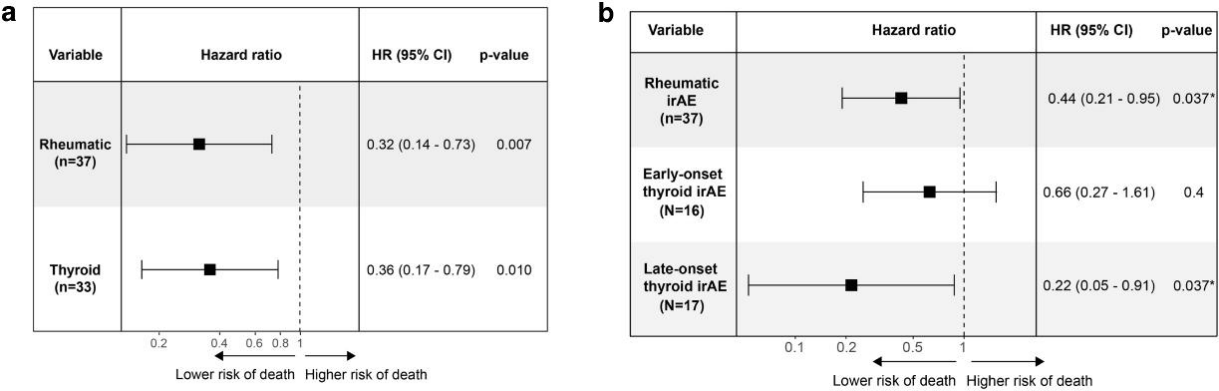
Rheumatic and late thyroid irAEs are associated with significantly better survival after adjusting for negative baseline factors and immortal time bias. (a) The survival benefit for rheumatic and thyroid irAE remained after adjusting for negative prognostic baseline factors using multivariate Cox regression. (b). The survival benefit for rheumatic and late, but not early, thyroid irAE also remained after adjusting for immortal time bias (time-dependent Cox-regression). *P* < 0.05 was considered significant and confidence intervals were set to 95%.

Next, we adjusted for the negative prognostic factors of worse ECOG performance status, elevated baseline LDH, and more advanced cancer stage (M1c). A significantly lower risk of death persisted for rheumatic irAE (HR: 0.32, 95% CI: 0.14–0.73, *P* = 0.007) and thyroid irAE (HR: 0.36, 95% CI: 0.17–0.79, *P* = 0.01) after adjusting for these baseline characteristics (Fig. 4a).

Finally, time-dependent Cox regression was used to adjust for immortal time bias. The development of rheumatic irAE was associated with a statistically significant reduction in the relative risk of death (HR: 0.44, 95% CI: 0.21–0.95, *P* = 0.037), and this reduction in risk was consistent over time. However, the univariate time-dependent analysis of thyroid irAEs was inconsistent over time. Therefore, two separate Cox regressions were performed, splitting the study period into two time windows: before and after 121 days (the median time to onset). In the early period (before 121 days), there was no significant difference in overall survival/risk of death between the thyroid and non-thyroid groups (HR: 0.66, 95% CI: 0.27–1.61; *P* = 0.4). In the late period (after 121 days), thyroid irAEs were significantly associated with a lower risk of death (HR: 0.22, 95% CI: 0.05–0.91, *P* = 0.037) (Fig. 4b).

### Survival outcomes in patients developing rare immune-related adverse events

**Figure 5:**
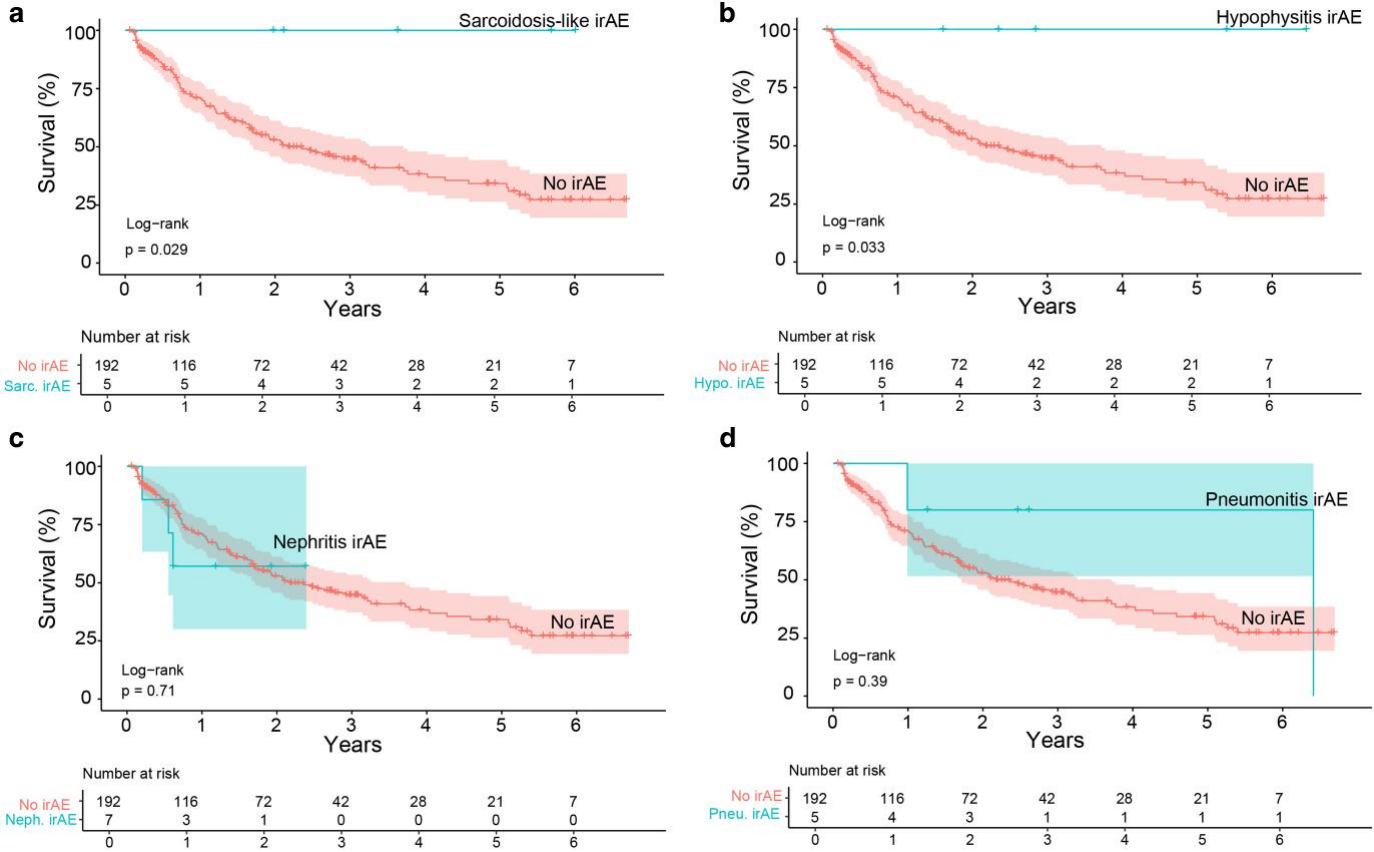
Survival associations in rare irAEs. Sarcoid irAE (a) and hypophysitis irAE (b) had superior survival compared to patients without irAE. No patients in these two groups died during the study period. There was no significant difference in survival between patients with nephritis irAE (c) or pneumonitis irAE (d) compared to patients without irAE. *P*-values were calculated with the log-rank test, where *P* < 0.05 was considered statistically significant. Confidence intervals (CI) for median survival were set to 95%. irAE: immune-related adverse events.

Nephritis, pneumonitis, sarcoidosis-like irAE and hypophysitis occurred in ≤2.5% of patients (Fig. 5a–d). Sarcoidosis-like irAE (Fig. 5a, *P* = 0.029) and hypophysitis (Fig. 5b, *P* = 0.033) were associated with superior survival whereas nephritis (HR: 1.24, 95% CI: 0.39–3.94, *P* = 0.71, Fig. 5c) and pneumonitis (HR: 0.54, 95% CI: 0.13–2.21, *P* = 0.39, Fig. 5d) were not. All patients who developed either sarcoidosis-like irAE or hypophysitis were still alive at the conclusion of the study. Due to the rarity of these irAE and lack of events (=death) in the sarcoidosis-like and hypophysitis groups, criteria to perform multivariate Cox regression and Time-dependent Cox regression were not fulfilled.

### Immune-related adverse events management and corticosteroid exposure in relation to overall survival

**Figure 6:**
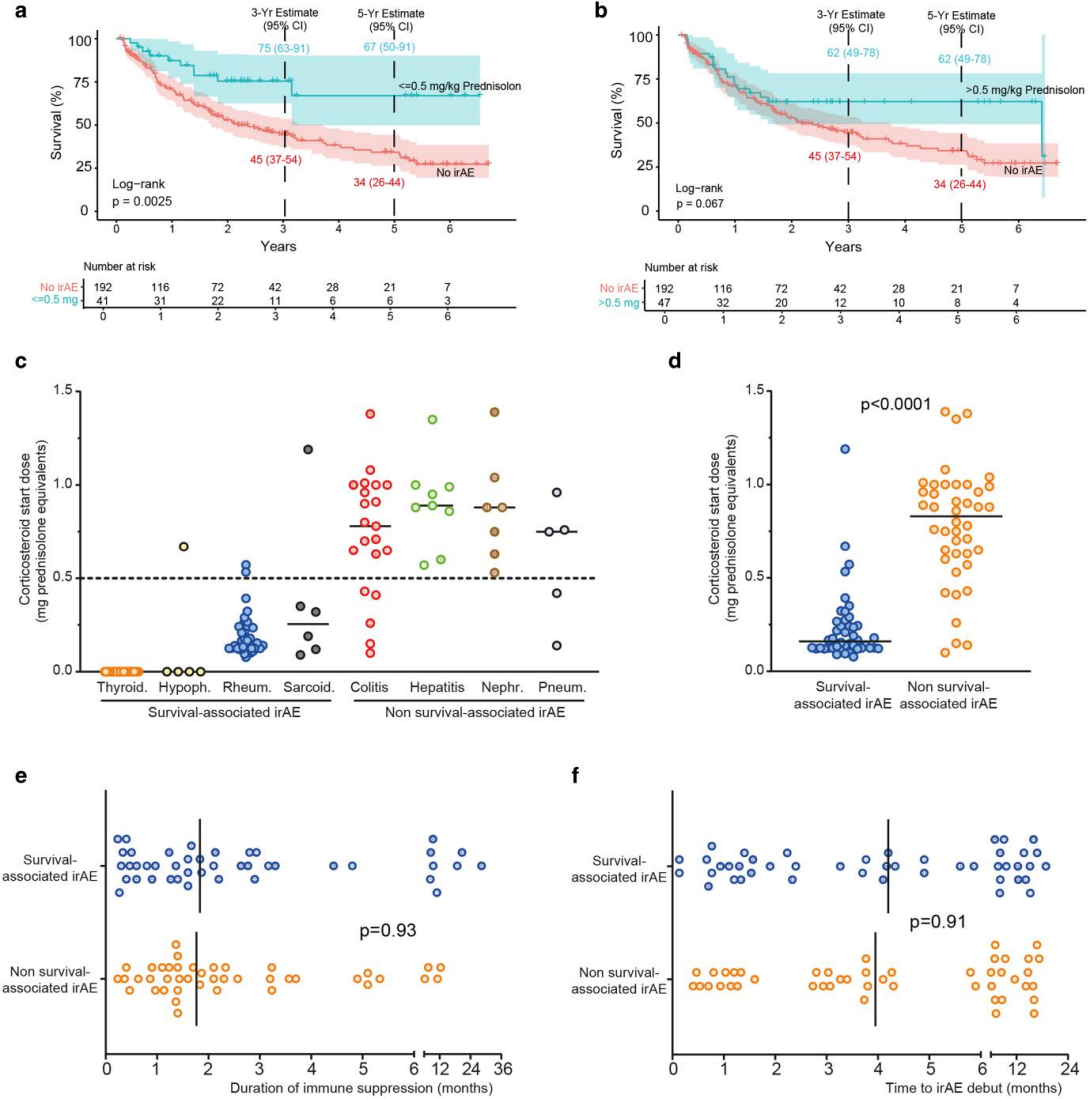
Survival associations and corticosteroid exposure. (a) Patients treated with low start dose corticosteroids (prednisolone < 0.5 mg/kg) had significantly better survival compared to patients with no irAE. (b), Patients treated with high starting dose corticosteroids (prednisolone > 0.5 mg/kg) had borderline significantly better survival compared to patients with no irAE. (c and d) The median start dose of corticosteroids was lower in patients with survival-associated compared to patients with non-survival-associated irAE. There was no difference in duration of immunosuppression (e) or time to debut of irAE (f) between survival-associated and non-survival-associated irAE. *P*-values were calculated with the log-rank test, where *P* < 0.05 was considered statistically significant (a and b). Confidence intervals (CI) for median survival were set to 95%. In (d-f), *P*-values were calculated using the Mann–Whitney test and *P* < 0.05 was considered significant. irAE: immune-related adverse events.

Among patients who developed irAEs, 84 received systemic corticosteroids, and 25 required endocrine hormone replacement therapy. Patients treated with a low initial corticosteroid dose (≤0.5 mg/kg) had a significantly longer median OS compared to patients without irAE (HR: 0.38, 95% CI: 0.20–0.73, *P* = 0.0025; Fig. 6a). Patients treated with a high initial corticosteroid dose (>0.5 mg/kg) also showed a trend toward improved survival (HR: 0.63, 95% CI: 0.38–1.04, *P* = 0.067; Fig. 6b).

Corticosteroids were not used for thyroid irAEs (0/33) and were rarely used for hypophysitis (1/5). Four of 37 patients (10.8%) with rheumatic irAEs required second-line immunosuppression—two with methotrexate monotherapy and two with a combination of methotrexate and TNF-α inhibition. Patients with survival-associated irAEs (rheumatic, thyroid, sarcoidosis-like, hypophysitis) were treated with significantly lower initial corticosteroid doses compared to those with non–survival-associated irAEs (colitis, hepatitis, nephritis, pneumonitis) (Fig. 6c and d). In contrast, the duration of corticosteroid therapy (Fig. 6e) and the time to irAE onset (Fig. 6f) did not differ between groups. Colitis showed a trend towards better survival in univariate analysis (*P* = 0.085) despite being treated with a high starting dose of corticosteroids. Therefore, we divided the colitis cohort into two groups with the median start dose of corticosteroids as cut off (0.8 mg/kg prednisolone equivalents). Both groups had numerically better survival than patients without irAE, but the difference was not significant (*P* = 0.36 for <0.8 mg/kg and *P* = 0.12 for ≥0.8 mg/kg) (Supplementary Figure S2).

## Discussion

This study demonstrates that rheumatic and late-onset thyroid immune-related adverse events (irAE) are clinical markers of superior overall survival (OS) in patients with advanced melanoma treated with PD-1 inhibition. Additionally, two rare irAEs, sarcoidosis-like irAE and hypophysitis irAE, were associated with exceptional survival. The frequency and types of most irAEs were consistent with previous reports on patients with melanoma treated with PD-1 inhibitors [11]. Rheumatic irAEs were more common in our cohort than in the initial clinical trials, an observation in agreement with other real-world studies [12, 13]. Three- and five-year survival rates in the present real-world cohort were nearly identical to those in the nivolumab arm of the CheckMate-067 study [14].

Our study shows that patients with rheumatic irAE have superior long-term survival. Rheumatic irAE was the most common irAE and occurred in 12% of patients, in line with previous reports [12, 13]. A subgroup of patients with rheumatic irAE required long-term corticosteroid and second-line immunosuppressive treatment. This suggests a prolonged immune activation, which is likely beneficial for a sustained immune response and agrees with the observation that rheumatic irAE is the most frequent chronic irAE (except for endocrine irAE) [15]. Notably, 10.8% patients with rheumatic irAE required second line immunosuppression compared to none with other irAE. The survival benefit suggests an immunophenotype that promotes both rheumatic irAE and an antitumor response. While the exact immune phenotype underlying development of rheumatic irAE remains undefined, data suggest involvement of both T and B cells. One study examined 20 patients with arthritis irAE and found a Th1-CD8+ T cell axis in both synovial fluid from inflamed joints and blood [16]. The authors speculate that the migration of CXCR1^high^ CD8⁺ T cells from the blood to the joints is mediated by the expression of CXCL9/10/11/16 by myeloid cells. Another study, including seven patients with arthritis irAE demonstrates that this group has a higher proportion of transitional B cells (CD19 + CD10 + CD24^high^CD38^high^) in peripheral blood compared to a group with no irAE [17]. The proportion of transitional B cells increased before the onset of overt arthritis irAE and decreased between the active and quiescent stages. Additionally, the study detected autoantibodies to joint-related type II collagen epitopes in 43% of arthritis-irAE patients, compared to none in non-irAE patients. Taken together, these studies suggest that arthritis irAE is associated with broad immune activation, involving T cells, B cells, and autoantibodies. It remains to be determined whether and how these mechanisms also promote a better antitumor response.

Another important finding of our study is the exceptional survival rate of patients who developed sarcoidosis-like irAE. Notably, none of the patients with sarcoidosis-like irAE died. Sarcoidosis-like irAE are characterized by the development of inflammatory, sarcoidosis-like granulomas in mediastinal lymph nodes and other locations, such as the lungs, skin, and bone [8]. Sarcoidosis-like irAEs are often asymptomatic and are typically detected on CT or PET/CT scans as enlarged mediastinal lymph nodes that strongly accumulate fluorodeoxyglucose. Consequently, sarcoidosis-like irAEs may be misinterpreted as cancer progression, but biopsies from affected tissues reveal sarcoidosis-like granulomas. Sarcoidosis-like irAE are rare, reported in 1–4% of PD-1 inhibitor-treated melanoma patients, which is in line with our data [18, 19]. In agreement with this study, there are previous reports indicating a superior treatment response in patients who develop sarcoidosis-like irAE. In 26 melanoma patients who developed sarcoidosis-like irAE, 71% had no evidence of progression over a median follow up of 11.5 months [9]. In a case-control study, patients with sarcoidosis-like irAE (*n* = 28) had significantly longer OS than patients with other irAE (*n* = 434) [20]. Another study reported higher objective response rates in melanoma patients receiving ICI treatment who developed sarcoidosis-like reactions [21]. In summary, our study agrees with previous observations that patients with sarcoidosis-like irAE have exceptional survival rates.

Thyroid irAEs were associated with improved survival in both univariate and multivariate analyses. However, when accounting for immortal time bias using time-dependent Cox regression, the survival benefit was only significant in patients who developed thyroid irAEs at 121 days or later. This suggests that early and late thyroid irAEs may reflect different biological mechanisms. Supporting this, no survival advantage was seen in patients treated with dual PD-1/CTLA-4 blockade, who developed thyroid irAEs earlier (median onset 60 days) compared with patients receiving PD-1 monotherapy (median onset 119 days) [22]. A meta-analysis of 47 studies similarly reported that overt hyperthyroidism tends to occur early, while subclinical disease and hypothyroidism develop later; notably, seven of these studies accounted for immortal time bias and still found a survival benefit [23]. Our study, with a larger cohort and longer follow-up, extends these findings by indicating that the survival benefit may be confined to late-onset thyroid irAEs.

As expected, hypophysitis was rare in this cohort of single PD-1 inhibitor-treated patients (5 out of 301 patients) but associated with excellent survival (all patients were alive at the data cutoff). Similarly, Jessel *et al* [24]. reported high response rates in patients with melanoma who developed PD-inhibitor-induced hypophysitis, 71% had a complete or partial response. Hypophysitis is much more common following CTLA-4 inhibition and confers a survival advantage in both dual PD-1 and CTLA-4 inhibition [22] and single CTLA-4 inhibition [25]. In this study, the median time to hypophysitis onset was 280 days, compared to 78 days in our previous study of dual ICI-treated patients [22]. The difference in time to onset is likely due to different biological mechanisms. CTLA-4 inhibitor-induced hypophysitis occurs through the direct binding of anti-CTLA-4 antibodies to CTLA-4 expressed on cells in the pituitary gland. These bound antibodies then induce a local complement activation which destroys pituitary cells [26]. The mechanisms behind PD-1 inhibitor-induced hypophysitis remain to be determined.

We found that patients treated with low-dose corticosteroids or hormone replacement for irAEs retained a significant survival advantage, whereas this was not observed in those receiving high-dose corticosteroids. This aligns with prior studies showing that early high-dose corticosteroid use for irAEs is associated with poorer survival in PD-1–treated melanoma patients [27, 28]. Similar findings have been reported with dual PD-1/CTLA-4 blockade, where a high peak corticosteroid dose—but not cumulative dose— and is linked to worse survival [29]. However, in our study, PD-1–treated patients with irAEs requiring high-dose corticosteroids, particularly colitis, still showed a borderline survival benefit. This suggests that corticosteroid dose alone does not fully explain survival differences and that irAE type is also important. Supporting this, a retrospective study of CTLA-4-induced hypophysitis showed superior survival with low-dose corticosteroids compared with high-dose treatment, although both groups had better survival than patients without hypophysitis [25]. The absence of a clear survival benefit for colitis in the present study may reflect the immunosuppressive effects of high-dose corticosteroids. Current guidelines recommend 1–2 mg/kg prednisolone for grade 3–4 colitis; however, in patients with good performance status, a lower initial corticosteroid dose could be considered for grade 3 colitis to avoid compromising antitumor immunity.

An important consideration is whether irAEs arise from single (PD-1) or dual (PD-1 plus CTLA-4) immune checkpoint inhibition. The present study includes only single-agent ICI, whereas our previous work examined dual ICI therapy [22]. Across studies, both similarities and differences were observed in survival associations and time to onset. As in the current study, rheumatic irAEs, hypophysitis, and colitis were associated with improved survival in patients receiving dual ICI. However, colitis and hypophysitis occurred earlier with dual ICI. Furthermore, colitis induced by CTLA-4 inhibition is more dependent on T helper cells and depletion of Tregs than PD-1 inhibitor-induced colitis [30, 31]. In contrast to colitis and hypophysitis, rheumatic irAEs had a similar time to onset in single vs dual ICI, supporting the notion that rheumatic irAEs are primarily PD-1–dependent [22].

A notable difference between single and dual ICI was observed for hepatitis. In this study, grade 2–4 hepatitis was uncommon (3%) and not associated with improved survival. In contrast, hepatitis was more frequent with dual ICI (13%) and correlated with superior survival [22]. Moreover, hepatitis occurred earlier (median 58 vs. 114 days) and was more often steroid-refractory in dual ICI-treated patients. Collectively, these findings suggest distinct immune mechanisms underlying hepatitis induced by single versus dual ICI. Notably, the immune response driving dual ICI-associated hepatitis may also promote durable antitumor activity that persists despite high-dose corticosteroids and the frequent need for second-line immunosuppression.

One limitation in our study is the retrospective, single-center study design, which may limit the generalizability of the results. However, this design has advantages as well. All patients are treated similarly by a small group of experienced physicians and nurses, according to ESMO guidelines for managing immunotherapy-related toxicities. Furthermore, immortal time bias is an inherent caveat in studies of the association between irAE and survival. IrAE is a time-dependent variable and superior survival in patients with irAE may reflect that they have survived long enough to develop irAE. In this study, we used time-dependent Cox regression to compensate for immortal time bias. Time-dependent Cox regression requires precise timing of events to yield correct results. Therefore, we used precisely timed events in our analysis. The exact start date of corticosteroids or hormone replacement therapy is recorded in electronic prescriptions made by the treating physician. This contrasts with lower grade irAE, which are not always recorded in patients’ medical records. The date of death is automatically recorded in patients’ electronic medical records and obtained from the Swedish Cause of Death Register, which is maintained by the National Board of Health and Welfare. Thus, our study design is optimized for performing time-dependent Cox regression. However, the exclusion of lower-grade irAE, which does not require systemic immunomodulation, may be a limitation because they are classified as non-irAE. This could lead to an underestimation of the survival benefit of certain irAE.

In conclusion, our study demonstrates that PD-1 inhibitor-treated patients who experience rheumatic, endocrine, and sarcoidosis-like irAE have superior survival and require lower doses of corticosteroids than non-survival-associated irAEs. Future research should focus on identifying the immune mechanisms behind survival-associated irAE. This knowledge could lead to the identification of new targetable immune mechanisms that could transform non-responders into responders. In addition, our findings support the exploration of lower-dose corticosteroid regimens in future prospective trials to optimize irAE management without compromising therapeutic outcomes.

## Supplementary Material

ltag004_Supplementary_Data

## Data Availability

The data that support the findings of this study are available on request from the corresponding author, ML. The data are not publicly available due to their containing information that could compromise the privacy of research participants.
