# Supplementary material for: PD-1 inhibitor-induced rheumatic, endocrine, and sarcoidosis-like immune-related adverse events in metastatic melanoma are associated with improved survival and lower corticosteroid exposure
Source: Immunother Adv. 2026 Feb 21;6(1):ltag004. doi: 10.1093/immadv/ltag004 (PMC12965203; doi:10.1093/immadv/ltag004)
Supplement: ltag004_Supplementary_Data [file ltag004_supplementary_data.pdf]

Supplementary Appendix

Contents

Table S1. Baseline characteristics………...2

Figure S1. Overall survival – all patients...3

Figure S2. Overall survival – colitis……..4

Supplementary table

Table S1. Baseline characteristics

	All patients n (%)	Any irAE n (%)	No irAE n (%)
**Age**			
**< 65**	96 (31.9)	33 (29.7)	63 (33.2)
**≥ 65**	205 (68.1)	78 (70.3)	127 (66.8)
**Sex**			
**Male**	173 (57.5)	62 (55.9)	111 (58.4)
**Female**	128 (42.5)	49 (44.1)	79 (41.6)
**BMI**			
**< 25**	87 (28.9)	34 (30.6)	53 (27.9)
**≥ 25**	179 (59.5)	64 (57.7)	115 (60.5)
**Unknown**	35 (11.6)	13 (11.7)	22 (11.6)
**Performance status (ECOG)**			
**0-1**	261 (86.7)	100 (90.1)	161 (84.7)
**2-4**	29 (9.6)	8 (7.2)	21 (11.1)
**Unknown**	11 (3.7)	3 (2.7)	8 (4.2)
**M-stage**			
**M0 (inoperable stage III)**	31 (10.3)	14 (12.6)	17 (8.9)
**M1a**	83 (27.6)	27 (24.3)	56 (29.5)
**M1b**	73 (24.3)	34 (30.6)	39 (20.5)
**M1c**	114 (37.9)	36 (32.4)	78 (41.1)
**LDH**			
**< 2 x ULN**	276 (91.7)	106 (95.5)	170 (89.5)
**≥ 2 x ULN**	21 (7.0)	5 (4.5)	16 (8.4)
**Unknown**	4 (1.3)	0 (0)	4 (2.1)
**BRAF-mutation**			
**Yes**	119 (39.5)	36 (32.4)	83 (43.7)
**No**	165 (54.8)	67 (60.4)	98 (51.6)
**Unknown**	17 (5.6)	8 (7.2)	9 (4.7)
**Previous systemic tx**			
**Yes**			
**No**	64 (21.3)	18 (16.2)	46 (24.2)
	237 (78.7)	93 (83.8)	144 (75.8)
**CRP**			
**< 5**	136 (45.2)	65 (58.6)	71 (37.4)
**≥ 5**	106 (35.2)	35 (31.5)	71 (37.4)
**Unknown**	59 (19.6)	11 (9.9)	48 (25.3)

BMI: Body-Mass index, PS (ECOG): performance-status (Eastern Cooperative Oncology Group), LDH: Lactate dehydrogenase, ULN: upper limit of normal (3.4 µkat/L), CRP: C-reactive protein.

Supplementary figure

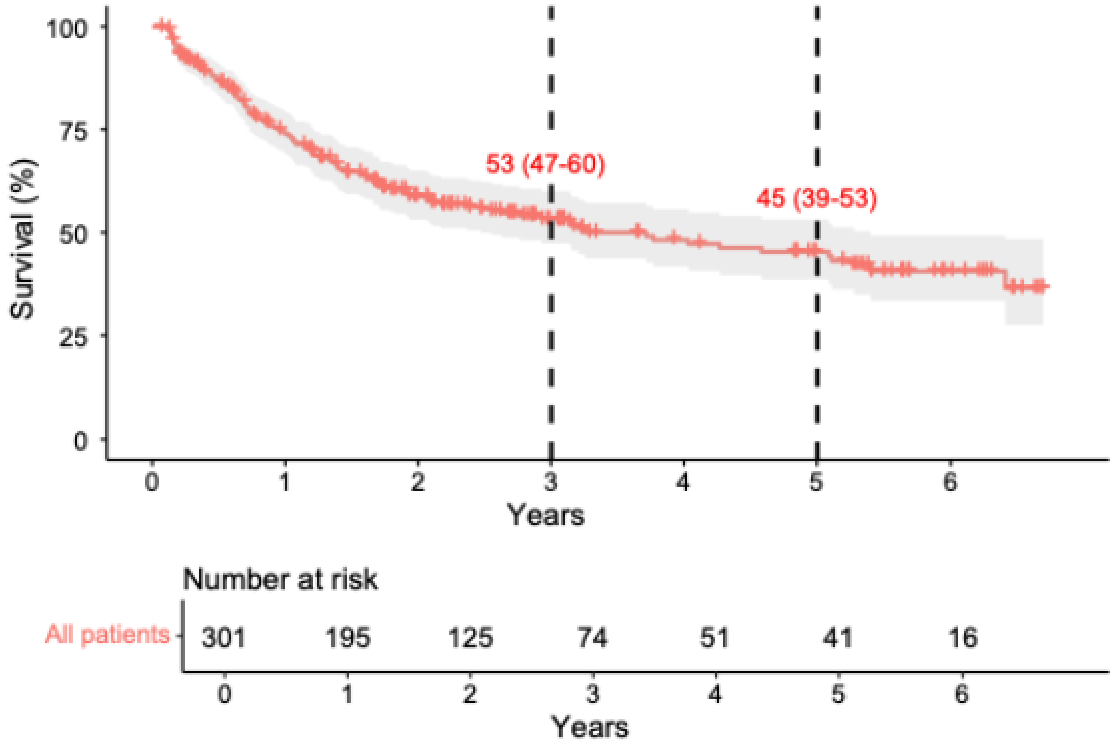

Figure S1. Overall survival – all patients.

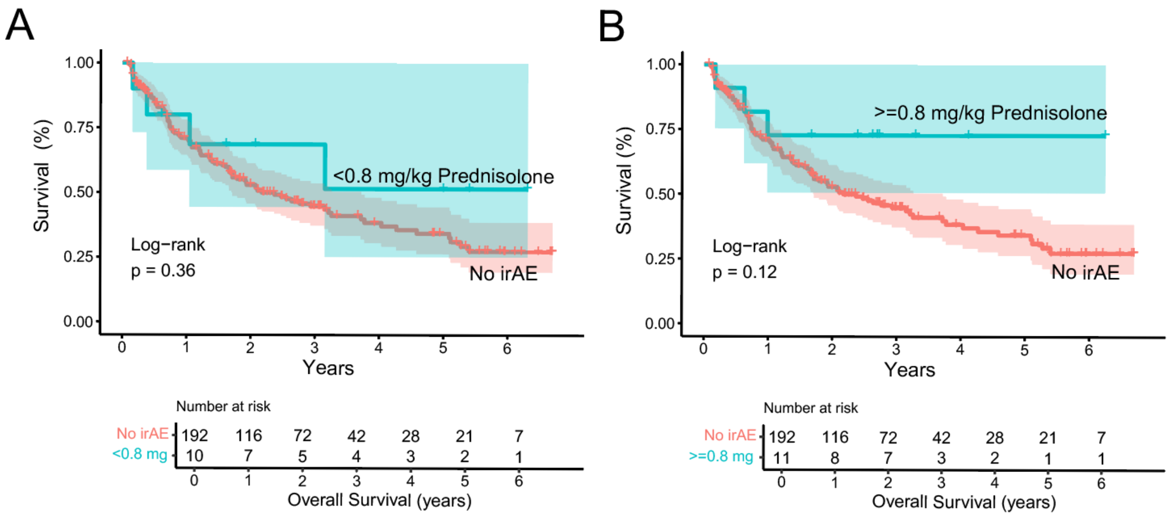

Figure S2. Overall survival – colitis

Patients with colitis irAE were divided into two groups based on the median start dose of corticosteroids; below median (<0.8 mg/kg prednisolone) or equal to or above median (>=0.8 mg/kg prednisolone). (A) Survival in patients (n=10) treated with <0.8 mg/kg prednisolone vs patients with no irAE. (B) Survival in patients (n=11) treated with >=0.8 mg/kg prednisolone vs patients with no irAE. P-values were calculated with the log-rank test, where p<0.05 was considered statistically significant. Confidence intervals (CI) for median survival were set to 95%. irAE: immune-related adverse events.
